# Some peace of mind: assessing a pilot intervention to promote mental health among widows of injecting drug users in north-east India

**DOI:** 10.1186/1471-2458-8-294

**Published:** 2008-08-22

**Authors:** Michelle Kermode, Alexandra Devine, Prabha Chandra, Bernice Dzuvichu, Thomhood Gilbert, Helen Herrman

**Affiliations:** 1 Nossal Institute for Global Health, University of Melbourne, Australia; 2 National Institute of Mental Health and Neurological Sciences, Bangalore, India; 3 Youth Action Resource Development (YARD), Dimapur, India; 4 Sneha Bhavan, Imphal, India; 5 ORYGEN Research Centre, University of Melbourne, Australia

## Abstract

**Background:**

HIV prevalence in north-east India is high and injecting drug use (IDU) is common. Due to HIV-related deaths there are increasing numbers of IDU widows, many of whom are HIV infected, and experiencing poor health, social isolation, discrimination and poverty, all factors likely to be compromising their mental health. There is increasing recognition of the links between HIV and mental health.

**Methods:**

The aim of this study was to pilot a peer-facilitated, participatory action group (PAG) process and assess the impact of the intervention on the mental health of participants. The intervention consisted of 10 PAG meetings involving 74 IDU widows. Changes in quality of life (WHOQOL-BREF), mental health (GHQ12) and somatic symptoms were assessed. The value of the intervention from the perspective of the participants was captured using a qualitative evaluation method (Most Significant Change).

**Results:**

Participants' quality of life, mental health and experience of somatic symptoms improved significantly over the course of the intervention, and the women told stories reflecting a range of 'significant changes'.

**Conclusion:**

This pilot intervention study demonstrated that a participatory approach to mental health promotion can have a positive impact on the lives of vulnerable women, and the potential to contribute to HIV prevention. Further investigation is warranted.

## Background

The north-east Indian states of Manipur and Nagaland are characterised by political instability, unemployment, and easy availability of heroin from across the Myanmar border. They are classified by the National AIDS Control Society [1] as high prevalence states for HIV, and intravenous drug use is an important route of HIV transmission [1,2]. The injecting drug user (IDU) population in these states constitutes 1.9–2.7% of the adult population [2]. In 2005, the HIV prevalence among IDUs in Manipur and Nagaland was reported to be 24% and 5% respectively, representing an increase in both states from the previous year [1]. However, much higher rates have also been reported: in a sample of IDUs from north-east India, 75% were found to be HIV positive [2]. Most IDUs in north-east India are men, an estimated 40% are married [3], and death rates have been high in the last five years, consequently the number of widows of IDUs has increased.

Widows in India are socially and economically disadvantaged, and the situation for widows of IDUs is often worse. They are frequently stigmatised on three levels – for being a woman, being a widow, and being HIV positive [4]. In 2004, a situation assessment of widows of IDUs conducted in Manipur found that they were faced with a range of psycho-social, economic and health problems. Many IDU widows and their children were HIV-infected and experiencing poor health, social isolation, grief, loneliness, discrimination, and poverty; all factors likely to be compromising their mental health [5]. Some widows reported engaging in HIV risk behaviours including alcohol and drug misuse, sex work and unprotected sex. Accessing HIV prevention services was not a priority for these women who were predominantly concerned about livelihood and their children's future [5].

Mental health is more than simply the absence of mental illness; it is the foundation for well-being and effective functioning for individuals and communities [6]. Mental illness is associated with indicators of poverty including low levels of education, poor housing and low income [7], and with other illnesses such as HIV infection [8]. Substance misuse, violence and health problems such as HIV and depression are more prevalent and more difficult to cope with in conditions of low income, limited education and unemployment [9].

The nexus between gender and mental health is well recognised. Socio-cultural beliefs about gender roles often diminish women's control over their own lives and restrict their access to economic resources and power within the society, and this in turn has a significant effect on their mental health status and the risk of mental illness especially depression [10-12].

The influence of gender on women's mental health is most evident in relation to depression. Globally, including in India, women are approximately twice as likely as men to experience depression and widows in India are more likely to suffer from mental illness than single or married women [10-12]. There is a need to promote the mental health of women in general and of widows in particular, both in India and further a field [12].

Mental health is associated with HIV in a range of ways. In relation to engagement in HIV risk behaviours, individuals with poor mental health as a group and including those with untreated mental illness and substance misuse problems, have a greater chance of exposure to HIV related risk behaviours. Many have less control over their lives than other populations, are more likely to find themselves in situations of risk, and have diminished ability to negotiate safe behaviours [13,14]. Interactions between drug and alcohol use and depression are common, and studies in India indicate that the former is associated with engagement in HIV risk behaviours, especially among those with mental health problems [15,16]. People living with HIV and AIDS have an increased risk of developing mental health problems including depression and substance misuse [13-15]. These conditions adversely affect HIV and AIDS treatment adherence, contribute to risk behaviours and exacerbate social difficulties associated with stigma and discrimination. These considerations raise the possibility that promoting the mental health of vulnerable groups may reduce the risk of engagement in HIV risk behaviours and thereby contribute to HIV prevention.

Emerging evidence indicates that mental health can be promoted by public health actions with vulnerable groups [6]. Just as physical health can be promoted, so too can mental health. A recent WHO report draws on a public health framework proposed initially by the Victorian Health Promotion Foundation [17] that identifies three key social and economic determinants of community and individual mental health: (1) social inclusion; (2) freedom from discrimination and violence; and (3) access to economic resources. This framework recognises that psychosocial and economic factors influence (protect or negate) a number of health-related behaviours such as substance misuse and risky sexual behaviours, that in turn affect all areas of health, including mental health [16,17].

While the health benefits of community participation are well understood in development work, health policy does not always reflect this, partly because the published evidence related to this approach is limited. Our intervention drew on participatory action research (PAR) approaches to health development that seek to empower target communities to actively identify problems and develop solutions in relation to particular research questions. This enhances their self-confidence and leadership skills, and assists them to address their own health and social needs [18,19]. For example, a study in Nepal demonstrated that community based participatory action had a significant positive impact on maternal and infant mortality [20]. Studies such as these and the one described in this paper help to narrow the evidence gap on the effectiveness of community participation to contribute to changes in health status.

This pilot intervention study began with the hypothesis that the implementation of structured and peer-facilitated participatory action groups (PAGs) among widows of IDUs in Manipur and Nagaland, with a focus on promoting mental health and well-being and informed by a strengths-based approach [21], would be associated with: (1) improved mental health; and (2) a reduced likelihood of engagement in HIV risk behaviours.

The objectives of the study were to: (1) learn about the women's perspectives on mental health and well-being and the links between mental health and HIV; (2) assess changes in the women's quality of life and mental health during the course of the intervention; (3) assess changes in engagement in HIV risk behaviours; (4) describe the process and outcome of the intervention from the perspective of the women; (5) document the process of establishing and conducting the intervention so it can be repeated or adapted in the future. This paper reports on the findings in relation to objectives 2, 3 and 4. A full description of the background, the intervention and the methods for this study has been published elsewhere [22], so the following methods section is brief.

## Methods

Six groups of IDU widows were established (three in each state) in mid 2006, with 9–16 widows in each group. The women were recruited through partnerships with local non-government organisations (NGOs) working in the field of HIV prevention. The NGOs contacted IDU widow's known to them, and through these women's networks, contacted other widows. All interested widows attended a meeting where the nature of the study and intervention were explained, and those women interested in participating were recruited. The districts covered by the participating NGOs were Imphal and Churachandpur in Manipur and Dimapur and Kohima in Nagaland. The intervention is described elsewhere [22] and more information is available from the authors on request. In brief, the intervention was based on the framework for mental health promotion [17], and consisted of ten peer-facilitated PAG meetings that were held every fortnight for half a day over a twenty week period. All meetings were participatory, strengths-based and comprised of a combination of structured activities and open discussion (Table 1). Two peer facilitators were trained and supported for each group, and were provided with flexible written guidelines for each session. The women's travel and childcare costs were covered, refreshments were provided, and the activities deliberately engendered fun and enjoyment for the women. Each group participated in an action planning process to develop strategies for promoting mental health and the sustainability of the groups. A range of quantitative and qualitative data was collected to assess the impact of the intervention on the lives of the women.

**Table 1:** Outline of the ten PAG meetings for widows of IDUs

**Session**	**Outline**	**Data Collection**
**1**	• Introduction to the PAG process	• Baseline questionnaires:
	• Identifying members' expectations	- WHO QOL-BREF
	• Highlighting individual strengths and skills	- GHQ12
		- Health Risk Questionnaire
		• Meeting summary report
**2**	• Concepts and determinants of mental health for widows of IDUs	• Focus Group Discussion
		• Meeting summary report
**3**	• Mental health and mental health promotion	• Meeting summary report
**4**	• Envisioning a positive future	• Meeting summary report
	• Promoting social inclusion	
**5**	• Addressing stigma and discrimination	• Meeting summary report
	• Relaxation techniques	
**6**	• Improving access to work and resources	• Meeting summary report
	• Prioritising ideas for action plan development	
**7**	• Developing action plans	• Meeting summary report
**8**	• Developing action plans	• Collection of MSC stories
	• MSC approach	• Meeting summary report
**9**	• Mental health and HIV	• Focus Group Discussion
	• Feedback of MSC stories	• Meeting summary report
**10**	• Finalising action plans	• Post-intervention questionnaires:
	• Celebration	- WHO QOL-BREF
		- GHQ12
		- Health Risk Questionnaire
		• Meeting summary report

### Quality of life, mental health, somatic symptoms and HIV risk behaviours

Three brief questionnaires were completed by the women during the first and last PAG meetings:

1. The short version of the WHO Quality of Life questionnaire (WHOQOL-BREF): Quality of life is a broad ranging concept and is assessed on a person's perceptions of various factors divided into four domains: (i) Physical health domain including pain and discomfort, energy and fatigue, mobility, sleep and work capacity; (ii) Psychological domain including spirituality, body image and appearance, thinking and learning and self-esteem; (iii) Social domain including personal relationships, sexual activity and social support; (iv) Environment domain including physical safety and security, physical environment (pollution, climate), financial resources, participation in recreation, home environment, transportation, and access and quality of health and social care [23,24]. Cronbach alpha values for each of the domain scores in the WHOQOL range from 0.71 to 0.86, demonstrating good internal consistency [25].

2. The General Health Questionnaire (GHQ12): The GHQ12 is a screening instrument for common mental disorders suitable for use in community, primary care and medical settings. It is used in many different countries and although the original version consisted of 60 items; shorter versions have been subsequently developed and validated [26]. The Cronbach's alpha for the GHQ12 is estimated to be between 0.82 – 0.86 [26]. Analysis of the GHQ12 data used a cut-off of three points, greater than three indicating potential presence of a common mental disorder such as depression or anxiety. The selection of this cut-off was based on findings from an earlier study using the GHQ12 in an Indian setting [27].

3. A Health and Well-being Questionnaire; Women in India who experience mental health problems such as depression often express their distress as somatic symptoms [28-30], so this questionnaire was adapted from an existing somatic symptom scale [31]. It asked participants to select how often they experience pain, various bodily sensations (e.g. weakness, trembling), disturbances of body functions (e.g. sleep, appetite), and reproductive symptoms (e.g. menstrual disturbance, vaginal discharge), as an indication of mental health. The second part of the questionnaire consisted of a small number of questions about sexual partners and drug and alcohol use adapted from a widely used Behavioural Surveillance Survey [32].

The WHOQOL-BREF and the GHQ12 are usually self-administered but assisted administration is possible for people with low literacy. All questionnaires were translated into the local languages, back translated and piloted with literate and non-literate women. The research teams and peer facilitators assessed the sensitivity and appropriateness of all questions before they were included in the study. Special attention was given to the more sensitive questions seeking information about sexual and substance use behaviours.

Data were analysed using SPSS 10. Individual assessment scores were summarised using frequency distributions and mean and standard deviations. Comparisons between baseline and follow up scores were done using paired t-test for continuous scores (WHOQOL-BREF and Health and Well-being Questionnaire) and McNamara Chi-square test for categorical variables (GHQ12). Data were analysed with the two states combined and separately.

### Value of the intervention from the participant and NGO perspectives

The Most Significant Change (MSC) method was used to evaluate the intervention from the perspectives of the participants and the partnering NGOs. MSC is a qualitative, participatory approach to monitoring and evaluation used in development projects [33]. In this study, we collected 'stories of change' from participants during the eighth PAG meeting, and involved different stakeholders in systematic selection of the stories that best captured what they considered to be the 'most significant change'. Reasons for selecting particular stories were also documented. The point of MSC is that both the content of the selected stories and the reasons for choosing them make the values of the different stakeholders explicit, and this can be used to foster dialogue between potentially competing perspectives, in this case that of the IDU widows and the NGOs. As the MSC approach tends to elicit positive stories (in relation to the intervention), this method is not used as an evaluation tool in isolation.

**Figure 1:**
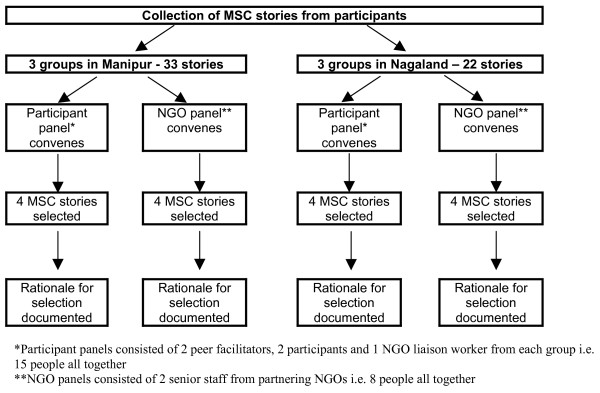
Process of Most Significant Change story selection.

Initially, stories of change were collected from all participants who were willing and able to provide them. The stories were told to the state-based research officers (BD, TG) in private and were recorded and subsequently transcribed and translated. Two panels were convened in each state: one participant panel consisting of two peer facilitators, two widows, and an NGO liaison worker from each group (three groups in each state), and one NGO panel consisting of two senior staff from each of four NGOs connected to the project. The story selection process involved reading all of the stories from the relevant state to the panel members who were given time to reflect on each story and then, through a collective process, select the four stories that, in their view, best represented the 'most significant change'. The two panels met and made their selections independent of each other (Figure 1). Panel members were encouraged to discuss the reasons why they selected these particular stories and this discussion was recorded, transcribed and translated. We were interested in the similarities and the differences between the stories selected by the participant and NGO panels, as well as similarities and differences in their reasons for selection. Additionally all of the stories were thematically analysed. This is an inductive approach that involves systematically coding and recoding the data in order to identify and organise both explicit and implicit patterns embedded in the data.

### Ethics and funding

This intervention study was funded by the United Kingdom's Department for International Development (DFID) through the Research and Learning Fund. Ethics approval was obtained from the University of Melbourne Human Research Ethics Committee (Australia) and the Emmanuel Hospital Association Institutional Review Board (India) in early 2006. Participation in the study was entirely voluntary, all participants provided informed consent and confidentiality was assured.

## Results

### Participation and demographics

Seventy-four women participated in the first PAG meeting, and 59 women completed the intervention (80% retention). The level of participation varied between NGOs (p = 0.04) and states (p = 0.002) (Table 2). The women were relatively young (mean age 32.5 years) and came from diverse ethnic and religious backgrounds (Table 3). The average length of widowhood was 4.3 years and the average number of children was 2.4. The majority of participants (81%) were not currently employed. Sources of income for those not formally employed included small businesses, support from family, sex work, and making handicrafts. Slightly more than half of the participants (53%) had completed secondary school and 30% had undergone some form of tertiary education. Nine percent of participants reported receiving no education. Although we did not systematically collect information regarding the participants' HIV status and drug use history, many of the women revealed their HIV positive status in the course of the intervention, and a small proportion had a history of injecting drug use.

**Table 2:** Retention in the intervention and reasons for dropping out

**Group**	**No. started**	**No. finished**	**Retention (%)**	**Reasons for attrition**
**Manipur 1**	16	14	87.5	• Attending detoxification program
				• Language barriers
**Manipur 2**	14	13	92.8	• Illness
**Manipur 3**	11	11	100	N/A
**Nagaland 1**	12	7	58.3	• Returned to home village
				• Death of participant
				• Death of children
				• Re-marriage
				• Drug use
**Nagaland 2**	9	5	55.5	• Ineligibility (not a widow)
				• Decided not to participate
				• Illness
**Nagaland 3**	12	9	75.0	• Childbirth
				• Illness

**TOTAL**	**74**	**59**	**79.7**	

**Table 3:** Demographics of the participants attending first PAG meeting

**Variable**	**Manipur n = 41**	**Nagaland n = 33**	**Total n = 74**
**Average age (range)**	33 yrs (23–46)	32 yrs (20–52)	32.5 yrs (20–52)
**Average length of widowhood (range)**	4.5 yrs (3 mth-12.8 yrs)	4.2 yrs (1 mth-11 yrs)	4.3 yrs (1 mth-12.8 yrs)
**Average no. children (range)**	2.2 (1–4)	2.7 (0–7)	2.4 (0–7)
**Ethnicity – n (%)**			
Naga	2 (5)	26 (79)	28 (38)
Meitei	26 (63)	0	26 (35)
Other	11 (27)	7 (21)	18(24)
Missing	2 (5)	0	2 (3)
**Religion – n (%)**			
Christian	14 (34)	30 (91)	44 (59)
Hindu	25 (61)	2 (6)	27 (37)
Muslim	0	1 (3)	1 (1)
Missing	2 (5)	0	2 (3)
**Employment – n (%)**			
Employed	2 (5)	10 (30)	12 (16)
Unemployed	37 (90)	23 (70)	60 (81)
Missing	2 (5)	0	2 (3)
**Education – n (%)**			
None	-	7 (21)	7 (9)
Primary	5 (12)	1 (3)	6 (8)
Secondary	15 (37)	24 (73)	39 (53)
Tertiary	21 (51)	1 (3)	22 (30)

### Changes in quality of life, mental health and somatic symptoms

In both states, all four Quality of Life domain scores increased across the course of the intervention indicating a general trend towards improvement in their quality of life (Table 3). The women experienced significant improvements in their physical and psychological health, and their interaction with the environment was more positive after participating in the intervention (p ≤ 0.05). While there was an increase in the social domain score, this did not reach statistical significance. The Quality of Life results in Manipur mirrored those for the group as a whole, but those in Nagaland were somewhat different as even though they all improved, none reached statistical significance (Table 4).

**Table 4:** Mean scores (SD) for Quality of Life domains at the beginning and end of the intervention by state

**Domain**	**Manipur (n = 38)**			**Nagaland (n = 21)**			**Total (n = 59)**		
	**Pre**	**Post**	**p value**	**Pre**	**Post**	**p value**	**Pre**	**Post**	**p value**

Physical	42 (15.1)	58 (13.7)	<0.01	55 (16.1)	57 (11.4)	NS	47 (16.7)	57 (12.8)	<0.01
Psychological	51 (18.5)	59 (15.5)	<0.01	53 (12.2)	57 (9.9)	NS	52 (16.5)	59 (13.7)	<0.01
Social	43 (20.3)	49 (23.2)	NS	52 (12.8)	53 (17.2)	NS	46 (18.3)	51 (21.2)	NS
Environmental	34 (18.1)	45 (12.1)	<0.01	45 (14.0)	47 (13.5)	NS	38 (17.5)	46 (12.5)	<0.01

The results from the GHQ12 indicate that almost three-quarters of the group (70%, 49/70) were possibly experiencing a common mental disorder such as depression or anxiety at baseline compared with 42% (24/57) at the end of the intervention (Table 4). This represents a significant decrease in the proportion likely to be experiencing a common mental disorder (p < 0.01). Similar improvements were noted in both states. Those who dropped out of the intervention compared to those who remained were no different at baseline in relation to their GHQ12 scores and a range of demographic measures (ethnicity, religion, education, employment and marital status).

In both states, the women experienced fewer somatic symptoms across the course of the intervention (as indicated by lower scores). However, pain was the only symptom that significantly decreased for the two states combined (p < 0.01).

In summary, these results indicate an overall improvement in several quality of life and mental health parameters across the course of the intervention, although the patterning of these improvements varied by state. Unfortunately, data were insufficient to conduct a complete analysis of the effect of the intervention on participants' engagement in HIV risk behaviours as too many of the respondents left the relevant questions blank. Only a handful of participants openly engaging in sex work responded adequately to these questions. This is discussed further in the Discussion section of the paper.

### Thematic analysis of the Most Significant Change stories

**Figure 2:**
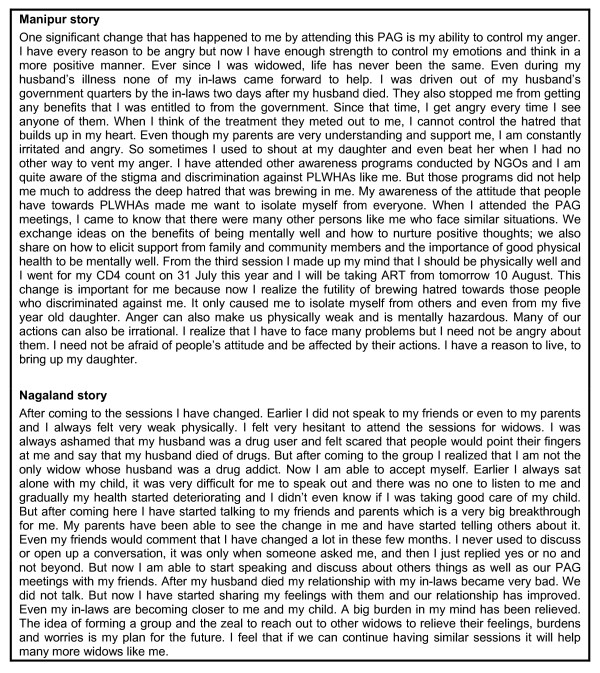
Two stories selected as representing the 'most significant change'.

Stories of change were collected from 33 participants in Manipur and 22 in Nagaland. Analysis of the stories uncovered a range of themes, most of which aligned with the socio-economic determinants of mental health i.e. social inclusion, freedom from discrimination, and economic participation. This is not surprising as the intervention was somewhat structured around these themes. Two additional themes were physical health and future orientation. A sample story from each state can be found in Figure 2.

#### Social inclusion

Many participants described how joining the group helped them overcome social isolation and provided them with a sense of belonging that was previously absent. The benefits of meeting and building relationships with other women in the same situation were frequently highlighted and the group became a source of nurturance and support for many of the women, and a stepping stone into the wider society for some.

I felt that others discriminated against me as my husband died from drugs. This made me ashamed and I never wanted to attend any social gatherings. I didn't want to interact with others and this resulted in depression and stress and a feeling that life had no value. Since I attended this meeting, it has encouraged me to interact socially. I have even begun to attend the church and other social gatherings. It has been good meeting other widows like me, who I never knew before, but now they have become my friends with whom I can share my problems. (Manipur 11)

A lot of participants gained confidence in their own abilities as a result of having to interact in a group setting.

Before I attended this PAG, I could never stand in a group and speak but during the PAG sessions I had many opportunities to share my opinion and speak in the group, which has helped me to realise some of my potentials. (Manipur 33)

Family conflict (mainly with in-laws) was one of the major sources of stress for the women. Many felt that the intervention had provided them with skills to better manage anger, thus enhancing their relationships with family.

My relationship with my family and in-laws has improved. Earlier I used to fight with them because I have very negative thoughts about them. Now I understand that this will only increase our worries and affect my mental health. I have been able to handle my anger. After I stopped shouting, I have observed that even my children have stopped fighting amongst themselves. This has helped me to strengthen my relationship with my family and friends as well. (Nagaland 1)

The PAG meetings deliberately incorporated activities that were fun such as games and singing, and this provided many of the women with a rare opportunity to experience joy and pleasure.

The fun and laughter during the PAG has also helped me a lot. It is only here that I experience fun and laughter. (Nagaland 20)

Finally, several of the participants said that they were inspired to help others.

I have been able to meet other people like me and have started thinking of helping other people like me as well. I have been able to learn how to access resources and this is helping me to manage my plans and finances better. I have already started sharing my experiences and benefits with other widows. Now I have **some peace of mind**. (Nagaland 12)

#### Discrimination

The theme of discrimination against people with HIV and AIDS generally, and IDU widows in particular, was evident in many of the women's stories. They described intense feelings of anger in relation to perceived discrimination from both family and society. Several of the women reported that learning to manage their anger allowed them to better cope with this source of adversity.

Prior to joining the PAG, I had a strong resentment fermenting inside me for a very long time. I would not be able to eat, sleep or do anything... nor take care of my children... People do not respect us because we were are widowed very young. They think that we do not have any other means of earning except by selling our body... Thinking about these things made my resentment grow from bad to worse. It has been about four months since I began attending this PAG meeting. I have realised that these problems will not go away... I can not hope nor expect any of them to change, but I can adjust with them. I need not brood over their attitude and let it affect my life and my relationship with my children. I can control my anger and seek my own course in life. Now I keep my priority concern on my children's welfare, so I've started attending the parents meetings at my children's school. (Manipur 20)

#### Economic participation

This theme was connected to both an appreciation of the allowance received as compensation for their travel and childcare costs, and a growing awareness of their collective ability to form self help groups for income generation.

Our allowances are very helpful because most of us are income-less at home. It is no longer necessary to demand money from the family as we can meet some of our needs now. With this allowance I buy Raja [tobacco] and sell it to get some profit... and I spend that on my other essential needs. With this allowance we have a sense of ownership in the family. (Manipur 1)

There are changes in my work also. I go to the agricultural field and forest to earn my livelihood. I was very shy and could not even think of doing something in the town. But now I have the confidence and I have started selling vegetables in the market and also go house to house. (Nagaland 9)

We do not have a voice, so we can become strong if we come together... We can start income generation and share our problems. If we are able to generate income it will help us. (Nagaland 13)

#### Improved physical health

Many women reported feeling much better physically and several had initiated contact with service providers as a result of participation in the group. Additionally, the relationship between physical and mental health was recognised by some women.

After coming to the meeting I realised that I am not alone and that has encouraged me to become more productive. I have also started accessing free medical services and medicines, which I never did even if I knew there were facilities available. I attended a free health camp for the first time last month. (Nagaland 11)

Even my physical health has improved in the last two to three months. I used to have severe gastric problems but now that has reduced. Being able to share and discuss my problems with friends has lightened my mental worries. (Nagaland 9)

#### Future orientation

Many of the women commented that the intervention contributed to renewed feelings of hope for the future and several appreciated the opportunity to create personal goals.

Before all my hope was gone but this meeting has given me new hope. I was like a dead soul, but now with this new hope I can carry on my life and my responsibility. This way it improves my mental health. (Manipur 11)

### Reasons for selecting stories

**Figure 3:**
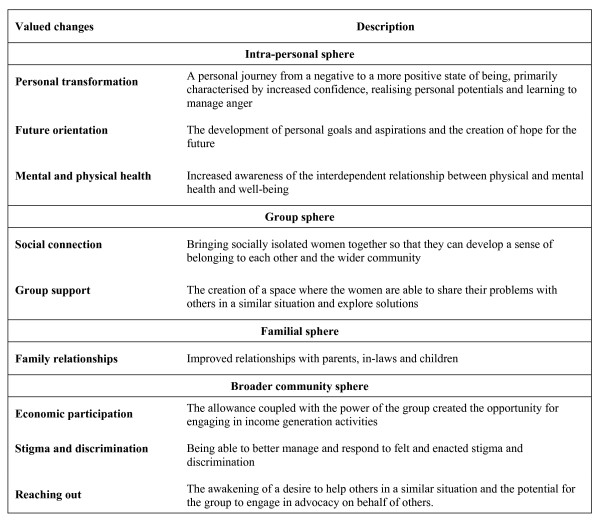
Categories of valued changes identified using the Most Significant Change method.

All stories were considered by the participant and NGO panels in each state, and each panel selected the four stories that best represented the 'most significant change'. The participant and NGO panels selected stories that were similar in thematic content, although the emphasis given to the reasons for the significance of the stories varied. The reasons the participant and NGO panel members give for selecting particular stories make explicit the type of changes valued by the different stakeholders. The emphasis given to different types of changes was reflected by the number of times they were mentioned in the course of the discussion. From the perspective of the storytellers, changes took place within themselves, and in relation to their interactions with the group, their families, and the broader community. The changes in these four spheres are summarised in Figure 3.

The participant panels particularly valued changes in the intra-personal, group and familial spheres, while the NGO panels tended to focus a lot more on the importance of changes located in the societal sphere.

## Discussion

This participatory intervention study to promote the mental health of widows of IDUs apparently had a positive impact on the widows' quality of life and their mental health. However, the sustainability of the improvement and the links between this and their engagement in HIV risk behaviours remain to be demonstrated.

While the physical, social and environmental domain scores of the quality of life measure improved significantly, the change in the social domain score did not. Qualitative findings, however, demonstrated improvements in social connectedness for the women. Had the sample sizes been larger, the change in the social domain scores may also have achieved statistical significance. For the women in Nagaland, the change in the scores for all four domains was not significant. The Quality of Life scores at baseline were higher among participants from Nagaland, and this together with the smaller sample size may account for the non-significant change observed in that state.

While the intervention's positive impact on mental health as assessed by the GHQ12 is encouraging, identifying a high proportion of the group (70%) as potentially having a common mental disorder (anxiety or depression) is a matter for concern. An overestimation of this proportion is possible for technical reasons. The GHQ12 includes questions about the presence of physical symptoms; as some of the women were HIV positive and experiencing physical illness, their resulting higher scores may have resulted in some instances of (mis)classification as 'cases'. On the other hand, the level of distress among this vulnerable group is evident to those familiar with their situation, and this finding is not surprising.

Given that women in India often express psychological distress as somatic symptoms [28,29], the observed reduction in the proportion of IDU widows reporting pain raises the possibility that this is connected to improved mental health, although the same improvements were not apparent in relation to the other somatic domains.

During the MSC process the participant and NGO panels gave differing emphasis to the reasons for selecting particular stories as representing the 'most significant change'. The former tended to value changes in the intra-personal, group and familial spheres, while the latter gave more focus to changes in the broader community sphere, perhaps reflecting their focus on sustainability of programs. This has implications for the design and evaluation of interventions. These will ideally meet the needs of both program participants and implementers if the contribution to HIV prevention is to be valued at all levels. The funding organisation is another stakeholder that could be involved in the MSC process, and they may well have a different perspective again. The MSC approach facilitates organisational learning for all connected to programs that aim to create change for participants.

The intervention generally, and the MSC process specifically, enhanced awareness among the NGO leadership of the struggles faced by IDU widows, especially those infected with HIV. Many of the NGOs providing HIV-related services in north-east India are male-dominated because the epidemic initially affected male IDUs predominantly. Prior to the intervention, IDU widows were isolated and powerless, and struggled to gain access to the few NGO programs relevant to them. Providing an opportunity for the widows to come together around the theme of mental health promotion has helped them to mobilise. Additionally, awareness of mental health as an important health issue for all people, including those with HIV infection, has increased.

While the number of women participating in the intervention was relatively small and the intervention relatively brief, these findings contribute to knowledge and understanding about the use of participatory interventions to improve the mental health of vulnerable women, and demonstrate the potential of this approach to contribute to HIV prevention.

The study has several limitations. As no control group met without receiving the structured program designed to promote mental health, we cannot know to what extent the observed improvements were related to the program content. The act of coming together may in itself have induced the changes. The attrition in some groups was disappointing but not unexpected given the stressful and at times unpredictable nature of the widows' lives, and an overall retention rate of 80% is high in this context. The linguistic and ethnic diversity and different literacy levels of the participants presented challenges during the intervention and data collection (see below). While most of these were overcome successfully with extra time, effort and research, we recommend that the participants in future groups be relatively homogenous. In relation to the intervention, we would also recommend that some HIV prevention education be included as even though these women's husbands had mostly died from AIDS, their level of HIV knowledge was poorer than expected. Extending the intervention to include training in advocacy and community mobilisation is also worth considering.

A relatively generous allowance was paid to the women to cover travel and childcare costs. This was important in motivating and recruiting women to participate in the intervention at the outset. While we do not know the extent to which the allowance influenced participation, six months after the last PAG meeting most of the groups are still meeting and some have expanded their activities. No allowance is now paid to the women, but support for the meetings is being given by NGOs.

Capturing sensitive information such as engagement in HIV risk behaviours proved to be challenging. North-east India is characterised by deeply felt conservative values, which are likely to have made it difficult for the widows to respond to questions about paid or unsafe sex, even though we had processes in place to assure confidentiality. The impact of improved mental health on engagement in HIV risk behaviours is therefore difficult to assess. Further work is required to develop sensitive and valid measures of sexual behaviour among groups such as these: including exploration of the women's perspectives on non-threatening methods for data collection about sexual behaviours, and trying other approaches such as participatory methods or individual narrative interviews with a trusted peer interviewer. Developing more effective methods for gathering information about sexual risk behaviours is essential for future research into the relationship between mental health and engagement in HIV risk behaviours.

Finally, the data collection tools had to be translated into the local language and back-translated. The meaning of individual questions in questionnaires such as the WHOQOL-BREF are nuanced and therefore not easy to exactly translate into languages that do not have the spectrum of words to communicate subtle differences in meaning. Imphal and Churachandpur in Manipur are only two hours apart by vehicle, but they do not share a common language, and two different translations were required for that state. Some people are able to read Manipuri when it is written in Manipuri script, while others can only read it in Bengali or transliterated Roman script. Nagamese is a spoken language with no official written form. Accurate translation of the data collection tools was an arduous task and further refinement and validation is warranted. This is typical of the many challenges encountered when trying to undertake quality research in remote settings.

## Conclusion

This pilot intervention study used a range of innovative approaches to program design, implementation and evaluation in order to reach a vulnerable group of women in a complex development setting with high HIV prevalence. The findings demonstrate that a participatory approach to mental health promotion can have a positive impact on the health and quality of life of vulnerable women. The intervention would benefit from further trialling and refinement and could be made available to other groups such as women living with HIV, the wives of IDUs, and specific sub-groups of widows such as sex workers and IDUs. Further research to evaluate the impact of the intervention on the lives of vulnerable women and to investigate the role of mental health promotion as a strategy for HIV prevention is warranted.

## Abbreviations

AIDS: Acquired Immune Deficiency Syndrome; DFID: Department of International Development; GHQ12: General health Questionnaire 12; HIV: Human Immunodeficiency Virus; IDUs: Injecting Drug Use/Users; MSC: Most Significant Change; NGO: Non Governmental Organization; PAG: Participatory Action Group; PAR: Participatory Action Research; WHO: World health Organization; WHOQOL BREF: WHO Quality of Life Abbreviated tool.

## Competing interests

The authors declare that they have no competing interests.

## Authors' contributions

HH, AD and MK were involved in conception of the study. HH, AD, MK and PC were involved in design, implementation and data analysis of the study. BD and TG were involved in the implementation and data analysis of the study. MK drafted the paper with contributions from AD, HH and PC. All authors read and approved the final manuscript.

**Table 5:** Proportion of participants with a possible common mental disorder as assessed by GHQ12 (using 3/4 cut-off) at the beginning and end of the intervention by state

**Manipur**			**Nagaland**			**Total**		
**Pre**	**Post**	**p value**	**Pre**	**Post**	**p value**	**Pre**	**Post**	**p value**

70%	49%	NS	70%	30%	0.04	70%	42%	<0.01
26/37	18/37		23/33	6/20		49/70	24/57	

**Table 6:** Somatic complaints at the beginning and end of the intervention

***Somatic symptom group***	*Manipur*			*Nagaland*			*Total*		
	**Pre ** **(SD)**	**Post ** **(SD)**	**p value**	**Pre ** **(SD)**	**Post ** **(SD)**	**p value**	**Pre ** **(SD)**	**Post ** **(SD)**	**p value**

Pain	8.6 (2.9)	7.4 (3.2)	NS n = 26	10.0 (1.7)	8.7 (2.4)	= 0.04 n = 16	9.3 (2.5)	7.9 (3.0)	<0.01 n = 42
Sensory sensations	16.9 (6.1)	15.6 (5.4)	NS n = 16	16.0 (4.7)	12.0 (3.5)	= 0.01 n = 8	16.7 (5.6)	14.5 (5.1)	NS n = 24
Bodily functions	7.0 (2.9)	6.2 (2.5)	NS n = 23	7.5 (3.6)	7.6 (1.9)	NS n = 8	7.1 (3.0)	6.6 (2.4)	NS n = 31
Reproductive health	3.3 (2.4)	5.4 (4.1)	= 0.02 n = 27	4.4 (2.7)	3.0 (2.5)	NS n = 11	3.7 (2.5)	4.7 (3.8)	NS n = 35

NS = not significant at p < 0.05.

## Pre-publication history

The pre-publication history for this paper can be accessed here:


